# Prognostic model of immune-related genes for patients with hepatocellular carcinoma

**DOI:** 10.3389/fsurg.2022.819491

**Published:** 2022-07-21

**Authors:** Qun Cai, Jinnan Duan, Liang Ding

**Affiliations:** ^1^ Department of Infectious Diseases and Liver Diseases, Ningbo Medical Center Lihuili Hospital, Affiliated Lihuili Hospital of Ningbo University, Ningbo, China; ^2^ Department of Infectious Diseases, Shaoxing People's Hospital, Shaoxing, China

**Keywords:** Immune-related genes, hepatocellular carcinoma, prognostic risk score, immunotherapy, predictor

## Abstract

**Background:**

Immune-related genes (IRGs) are closely connected to the occurrence and development of tumors. Their influence on the prognosis of patients with HCC, however, remains unclear.

**Methods:**

From the TCGA database, we integrated 365 liver cancer tissues and 50 normal tissues to identify differential immune genes related to prognosis. Multivariate COX analysis was used to establish a new prognostic index on account of IRGs, whereby risk score = (Expression level of HSPA4*0.022) + (Expression level of PSMD14*0.042) + (Expression level of RBP2*0.019) + (Expression level of MAPT*0.197) + (Expression level of TRAF3*0.146) + (Expression level of NDRG1*(0.006) + (Expression level of NRAS*0.027) + (Expression level of IL17D*0.075).

**Results:**

The risk score was clearly correlated with an unfavorable survival rate and with clinical characteristics. By integrating the immune-related risk score model with clinical features, a nomogram was constructed to predict the survival rate of HCC patients (1-, 3- and 5-year AUC of 0.721, 0.747 and 0.781, respectively).

**Conclusion:**

We have established a valuable prognostic risk score for HCC patients that may be a better predictor of survival than the present method. With the risk score's strong predictive value for immune cells and functions, it may provide clinical guidance for the diagnosis and prognosis of different immunophenotypes, and provide multiple therapeutic targets for the treatment of HCC patients based on subtype-specific immune molecules.

## Introduction

Hepatocellular carcinoma (HCC) is the second prime cause of cancer-related deaths internationally (1). Approximately 782,500 new cases of HCC are diagnosed worldwide each year (2); poor prognosis remains the most common feature in HCC patients due to their late diagnosis (3). HCC has several known risk factors, including hepatitis viral infection, alcohol insobriety, autoimmune hepatitis, adiposis and grievous metabolic diseases (4). The occurrence of HCC is closely related to an inflammatory microenvironment caused by virus infection, obesity and other liver damage (5). Tumor-associated immune cells are the main component of the inflammatory microenvironment of liver cancer. These cells respond to environmental signals and participate in the development of tumors (6).

Current clinical treatment options for HCC are varied and include surgical resection, liver transplantation, targeted therapy and immunotherapy. However, the prognosis of patients with liver cancer remains far from satisfactory (7). Consequently, the confirmation of prognostic biomarkers and novel drug targets is essential in providing HCC patients with better prognostic information and more efficient personalized treatments (8). Immune-related genes (IRGs) play a key role in regulating the systemic immune response (9). Improving our knowledge of IRGs is critical to understanding the mechanism of cancer immunotherapy. Some key IRGs could also serve as biomarkers to predict the outcome of cancer patients (10).

The aims of this research are to evaluate the prognostic significance of IRGs in HCC, to construct an IRG-based prognostic score, and to investigate the possible mechanism by which these immune genes affect the development of liver cancer. Ideally, the predictive scoring model could help clinicians estimate the risk of HCC in clinical practice and provide important information for personalized immunotherapy of HCC.

## Methods

### TCGA data and IRG identification

Transcriptome RNA sequencing data and clinical information for HCC was downloaded from the TCGA database (https://cancergenome.nih.gov/). There were 374 liver cancer tissue samples and 50 normal liver tissue samples. Overall, 365 patients were included, while HCC patients with zero overall survival were excluded. IRGs were identified from the ImmPort database (https://www.immport.org/), with 2,483 genes included in this study.

### Differentially expressed IRG

The R LIMMA software package (FDRFilter = 0.05, logFCFilter = 1 and Wilcox Test) was used to screen for differentially expressed genes between liver cancer and normal liver tissues. Data for these differentially expressed IRGs was then extracted. The R heatmap software package was used to display data as heat maps and volcano maps.

### Construction and validation of IRG-based prognostic model

To estimate the relationship between IRG expression and the overall survival rate of patients, we applied the univariate Cox proportional hazard regression analysis. A predictive model for IRGs was constructed depending upon their expression and the contribution coefficient (*β*) from the multivariate Cox proportional hazard regression model. The risk score was calculated for each patient and used to classify them into two groups (low- and high-risk) based on the optimal cut-off value for gene expression. The ICGC database (243 HCC samples) was applied to validate the precision of the risk score model.

### The relationship of Risk score with clinical information and immune cells

**Figure 1:**
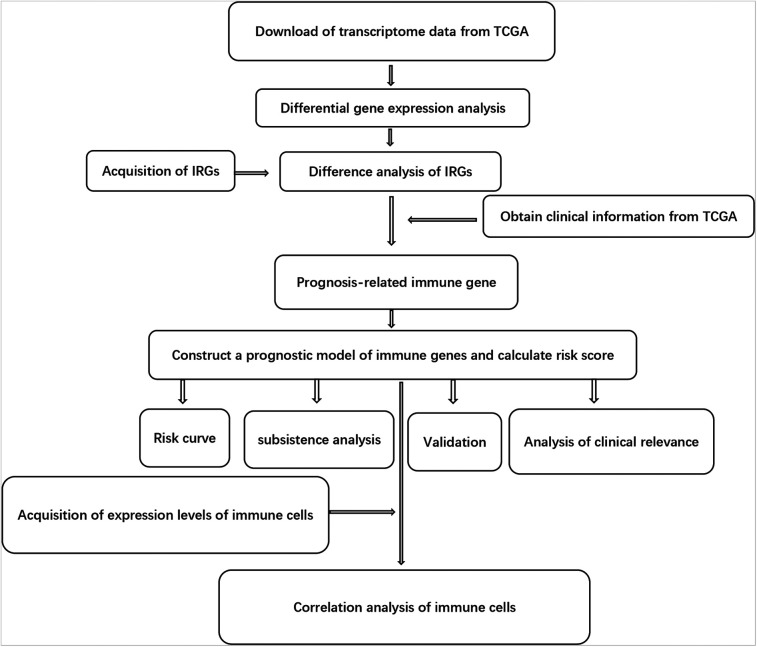
The flow chart of this study.

The clinical information available included gender, age, survival time, AJCC (American Joint Committee on Cancer) tumor grade and TNM (Tumor, Node and Metastasis) staging system. Tumor-infiltrating immune cells involved B cells, CD4 T cells, CD8 T cells, macrophages, neutrophils and dendritic cells. The TIMER online database analyzed and visualized the number of tumor-infiltrating immune cells (https://cistrome.shinyapps.io/timer/). We also used the Tumor Immunity Estimation Resource to analyze the correlation between risk score and 6 immune infiltrating cell types. The purity-corrected partial Spearman's correlation coefficient was used to evaluate the relationship between them. The flow chart for this research is shown in Figure 1.

### Construction and verification of the nomogram

Employing clinical information from the TCGA-LIHC cohort, multivariate Cox regression analysis was used to identify significant clinical variables. The nomogram was constructed using the R “rms” package combining the clinicopathological characteristics and risk scores of the patients. The ROC curve was used to evaluate the nomogram's 1-, 3- and 5-year prognosis, and the calibration curve was constructed by the bootstrap method (1,000 cycles) displaying the deviation between the predicted values and the actual probability of occurrence.

### Statistical analysis

Statistical analyses were performed using R software (version 3.6). Pearson correlation coefficient was used to evaluate correlations among genes in coexpression analysis. The patients were divided into high or low group according to the median expression of the gene in the TCGA and ICGC database, respectively. Kaplan–Meier curves were constructed and the log-rank test was performed using R package survival software. Univariate and multivariate Cox regression analyses were used to determine independent prognostic factors. ROC analysis with area under the curve (AUC) was used to evaluate the accuracy of the Risk score. The nomogram was developed by incorporating the clinicopathological features and risk scores of patients using the R “rms” package. A *P*-value of <0.05 indicated statistical significance.

## Results

### Identification of differentially expressed IRG

**Figure 2:**
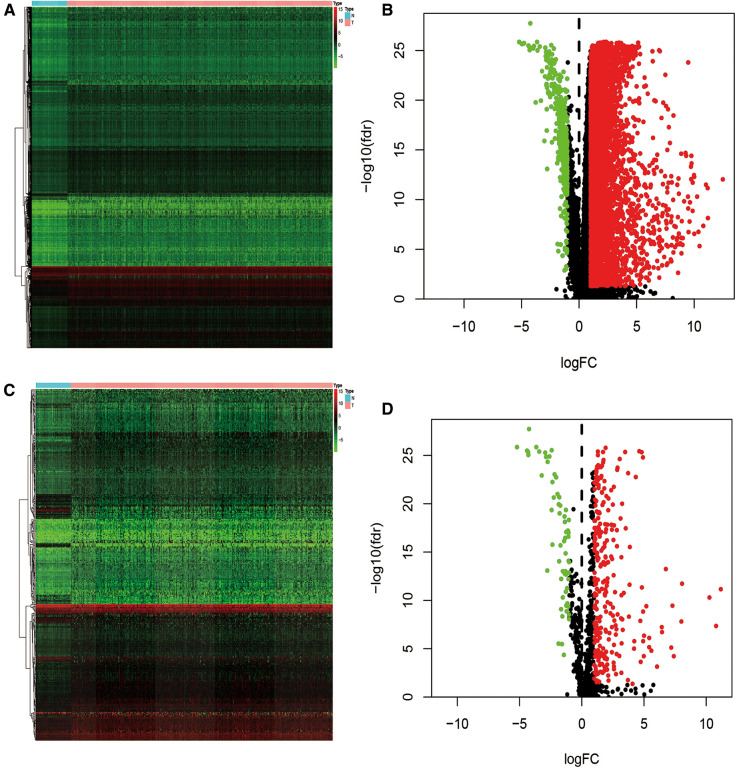
Heatmap and volcano plot of DEGs (**A,B**) and IRGs (**C,D**) between normal and HCC tissues.

Data from the TCGA database was extracted from 365 cases of liver cancer and 50 cases of non-diseased liver. 7,667 differentially expressed genes (DEG) were verified between the two groups, including 7,273 up-regulated and 394 down-regulated genes (Figures 2A,B). We then cross-matched these DEGs against the list of IRGs to identify differentially expressed immune genes. Among the total DEG, we identified 335 differentially expressed IRGs, of which 267 were up-regulated and 62 were down-regulated (Figures 2C,D).

### Construction of risk score model

**Figure 3:**
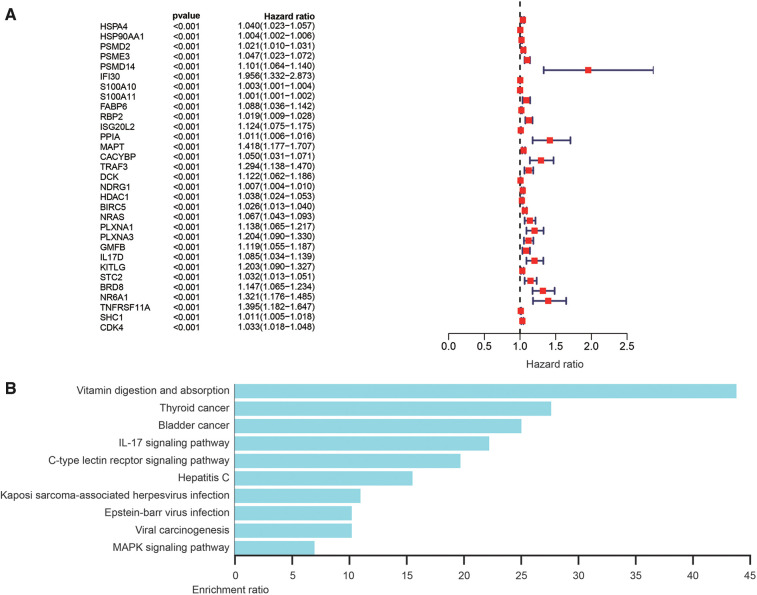
Analysis of survival-related differentially expressed IRGs. (**A**) Hazard ratio forest chart shows the predicted value of IRGs. (**B**) Analysis of KEGG pathway of survival-related IRGs.

The most important indicator of prognosis is patient survival rate. Accordingly, we investigated for associations between IRG expression and patient survival using univariate Cox proportional hazard regression analysis. The results showed that 31s IRG were remarkably related with the survival of HCC patients (Figure 3A). We also conducted enrichment analysis of the KEGG pathways for these 31 IRGs (Figure 3B). Cox regression analysis of these genes was applied to construct a risk score model. The calculation formula for the risk score contained eight IRGs, the coefficients and HR values of which are shown in Table 1. The calculation formula for the Risk score is as follows: Risk score = (Expression level of HSPA4*0.022) + (Expression level of PSMD14*0.042) + (Expression level of RBP2*0.019) + (Expression level of MAPT*0.197) + (Expression level of TRAF3*0.146) + (Expression level of NDRG1*(0.006) + (Expression level of NRAS*0.027) + (Expression level of IL17D*0.075).

**Table 1:** Prognostic-related immune genes obtained through Cox regression analysis.

IRGs	Coef.	HR	HR.95L	HR.95L	*p* value
HSPA4	0.022	1.023	1.003	1.042	0.021
PSMD14	0.042	1.043	0.994	1.091	0.089
RBP2	0.019	1.020	1.008	1.031	0.001
MAPT	0.197	1.218	0.987	1.504	0.066
TRAF3	0.146	1.157	0.991	1.352	0.065
NDRG1	0.006	1.006	1.003	1.009	0.000
NRAS	0.027	1.028	0.997	1.059	0.076
IL17D	0.075	1.078	1.023	1.135	0.005

### Evaluation and validation of risk score performance

**Figure 4:**
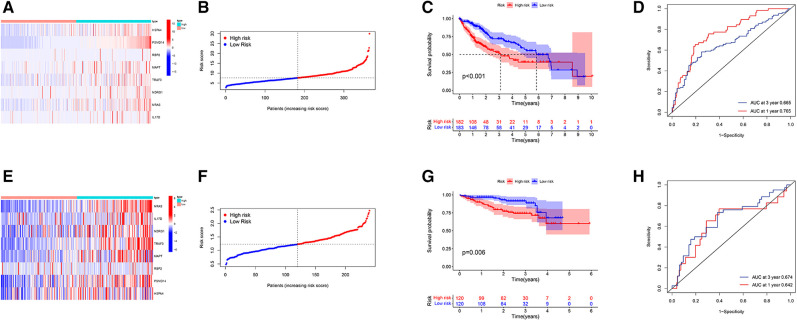
Prognostic evaluation and validation based on IRG risk score in TCGA-LIHC and ICGC-LIHC database. (**A**) Heatmap of gene expression profiles in high-risk and low-risk populations. (**B**) The grade and distribution of prognostic indicators for high-risk and low-risk populations. (**C**) Comparison of survival time between high-risk and low-risk groups. (**D**) Risk score for predicting the 1- and 3-year overall survival (OS) rates of HCC patients. (**E**) Heatmap of gene expression profiles in high-risk and low-risk populations. (**F**) The grade and distribution of prognostic indicators for high-risk and low-risk populations. (**G**) Comparison of survival time between high-risk and low-risk groups. (**H**) Risk score for predicting the 1- and 3-year OS rates of HCC patients. (**A–D** from the TCGA-LIHC database and **E–H** from the ICGC-LIHC database).

HCC patients were divided into a high-risk group (risk score ≥7.65) or low-risk group (risk score <7.65) according to the median in TCGA-LIHC. The difference in IRG expression profiles between the two groups is demonstrated in Figure 4A, and risk score ranking is demonstrated in Figure 4B. The mortality rate observed for the high-risk group was remarkably higher than for the low-risk group (Figure 4C), while the AUROC of the risk score for 1- and 3-year mortality were 0.765 and 0.665, respectively (Figure 4D). The risk score was validated using the ICGC database, which includes 243 LIHC samples, HCC patients were divided into a high-risk group (risk score ≥ 1.24) or low-risk group (risk score <1.24) according to the median. The difference in IRG expression profiles between the two groups is demonstrated in Figure 4E and risk score ranking is demonstrated in Figure 4F. The mortality rate observed for the high-risk group was remarkably higher than for the low-risk group (Figure 4G); the AUROC of the risk score for 1- and 3-year mortality were 0.642 and 0.674, respectively (Figure 4H).

### Construction and verification of the nomogram

**Figure 5:**
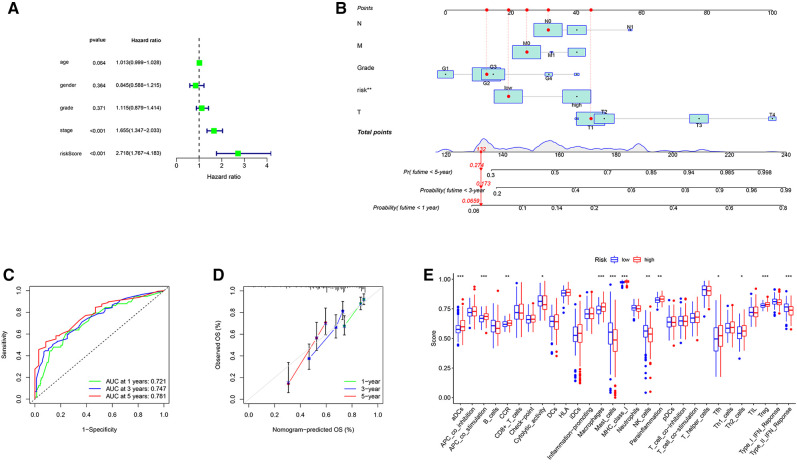
Construction of the nomogram and analysis of ssGSEA scores. (**A**) Results of the multivariate Cox regression analyses regarding OS in the TCGA cohort. (**B**) The nomogram for predicting 1-, 3- and 5-years overall survival in TCGA-LIHC cohort. (**C**) ROC analyses of the nomogram predicting of 1-, 3- and 5-years overall survival. (**D**) Calibration curve for validation of the nomogram. (**E**) Comparison of the ssGSEA scores between different risk groups in the TCGA cohort.

With clinical information from the TCGA-LIHC cohort, multivariate Cox regression analysis was used to identify significant clinical variables (Figure 5A). A nomogram was constructed by integrating risk scores and clinicopathological features to predict 1-, 3-, and 5-year overall survival in HCC patients (Figure 5B). The AUCs were 0.721, 0.747 and 0.781 for predicting in 1-, 3- and 5-years OS, respectively (Figure 5C). The calibration curve shows that the predicted and actual results of our nomogram are significantly consistent (Figure 5D).

### Relationship between IRG-based Risk score and clinical features

**Figure 6:**
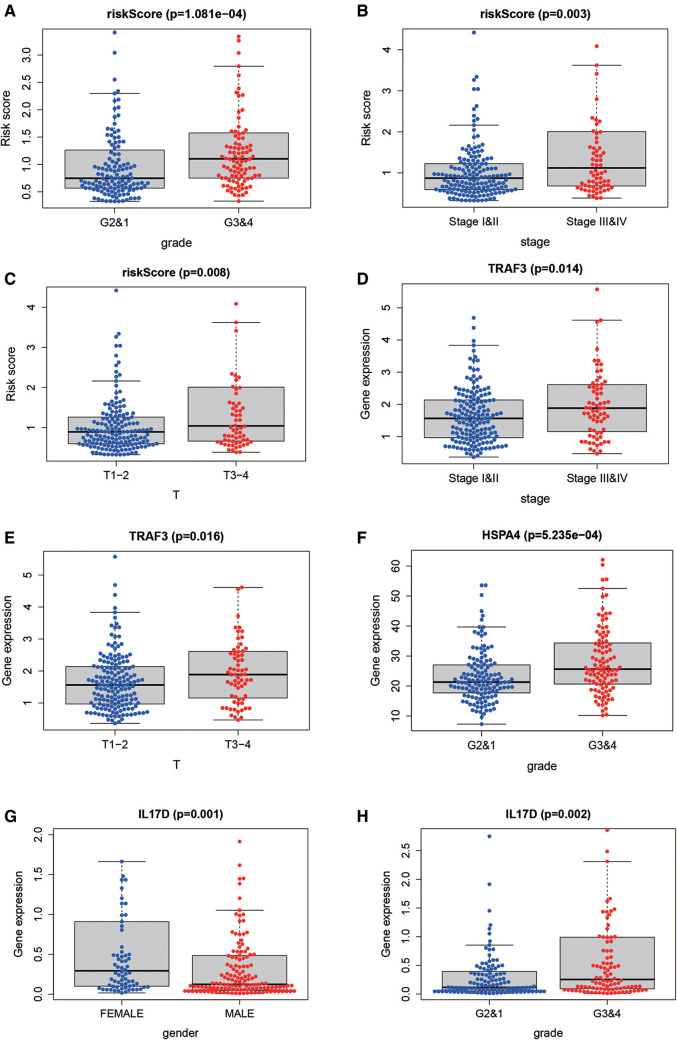
The correlation between risk score and clinical information. The risk score showed significant positive correlation with liver cancer patient grade (**A**), stage (**B**) and TNM stage (**C**). The risk score-derived immune gene TRAF3 showed significant correlation with stage and TNM (**D,E**), HSPA4 with grade (**F**), IL17D with gender and grade (**G,H**).

**Figure 7:**
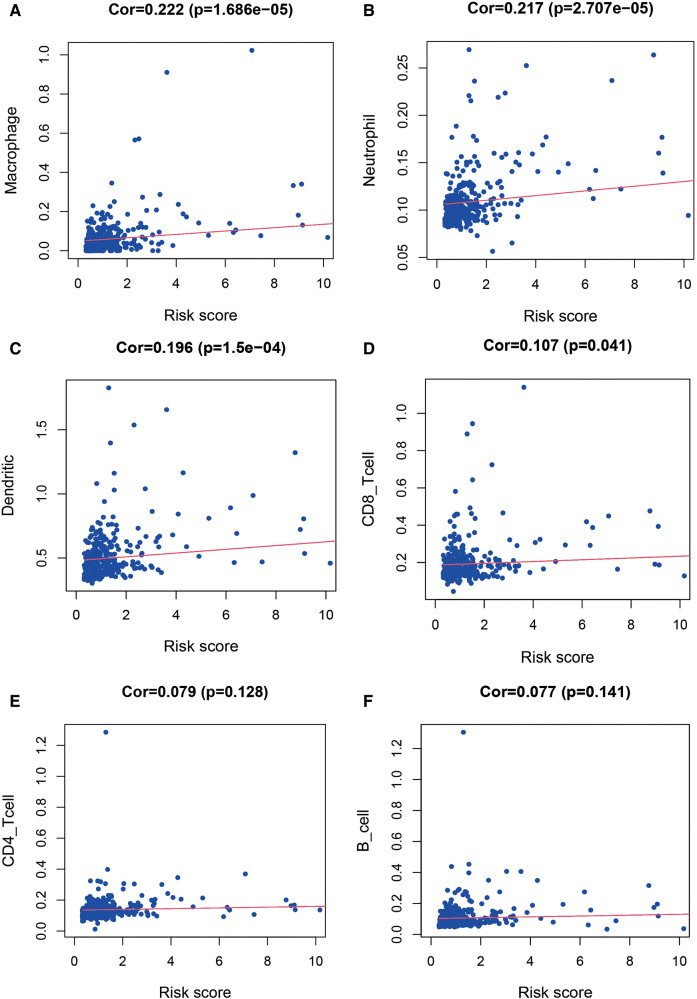
Relationship between the risk score and infiltration abundance of six immune cells. We further analyzed the correlation between risk score and immune cell infiltration. The number of macrophages (**A**), neutrophils (**B**), dendritic cells (**C**) and CD8 T cells (**D**) showed significant positive correlation with risk score, whereas CD4 T cells (**E**) and B cells (**F**) did not show any significant correlation.

To further explore the correlation of risk scores with immune status, we quantified enriched fractional subsets and associated functions of different immune cells by ssGSEA, including 16 immune cells and 13 immune-related function analyses. Interestingly, there were significant differences in both immune cell subsets and cellular function between the high- and low-risk groups. The scores for type-II IFN response, mast-cells and natural killer cells were lower in the high-risk group, while those for the macrophages, Tregs and Th2-Cells showed an opposite trend (Figure 5E). Univariate and multivariate Cox analyses were used to evaluate the prognostic value of the risk score (Table 2). We further analyzed the correlation between the IRG-based risk score and clinical information. The risk score showed significant positive correlations with liver cancer patient grade, AJCC stage and TNM stage. The risk score-derived immune gene HSPA4 showed significant correlation with grade, IL17D with gender and grade, and TRAF3 with stage and TNM (Figure 6). We further analyzed the correlation between risk score and immune cell infiltration. The number of CD8 T cells, dendritic cells, macrophages and neutrophils showed significant positive correlation with risk score, whereas CD4 T cells and B cells did not show any significant correlation (Figure 7).

**Table 2:** Independent prognostic analysis of clinical information and risk score.

Variables	Univariate analysis OR (95% CI)	*p* value	Multivariate analysis OR (95% CI)	*p* value
Age	1.001 (0.987–1.024)	0.588	1.014 (0.994–1.034)	0.174
Gender	0.781 (0.488–1.250)	0.303	1.222 (0.708–2.109)	0.471
Grade	0.740 (0.370–1.478)	0.393	0.717 (0.350–1.469)	0.364
Stage	1.864 (1.455–2.387)	0.000	0.940 (0.345–2.563)	0.904
T	1.803 (1.433–2.269)	0.000	1.818 (0.736–4.489)	0.195
M	3.847 (1.206–12.272)	0.023	1.925 (0.492–7.531)	0.347
N	2.020 (0.494–8.270)	0.328	2.341 (0.389–14.085)	0.353
Riskscore	1.172 (1.123–1.223)	0.000	1.162 (1.107–1.220)	0.000

### Transcription factors and regulatory networks

**Figure 8:**
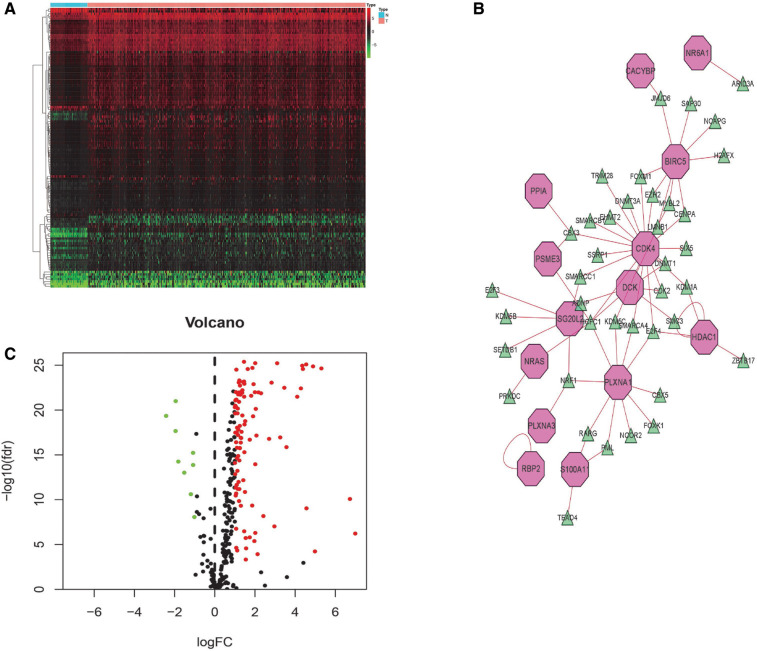
TF-mediated regulatory network. Heatmap (**A**) and volcano (**B**) plot of differentially expressed TFs. (**C**) Construct a regulatory network based on differentially expressed TFs and survival-related IRGs (green triangles indicate TFs and pink polygons indicate IRGs).

Transcription factors (TFs) directly control gene expression levels. A total of 318 TFs were present in the Cistrome Cancer database (http://cistrome.org/). Comparison of the two risk score groups (high and low) revealed 117 differentially expressed TFs. Cytoscape (V3.6.1) was used to assemble regulatory networks of the survival-related IRGs for these TFs (Figure 8).

### Further validation of the IRG-based Risk score

**Figure 9:**
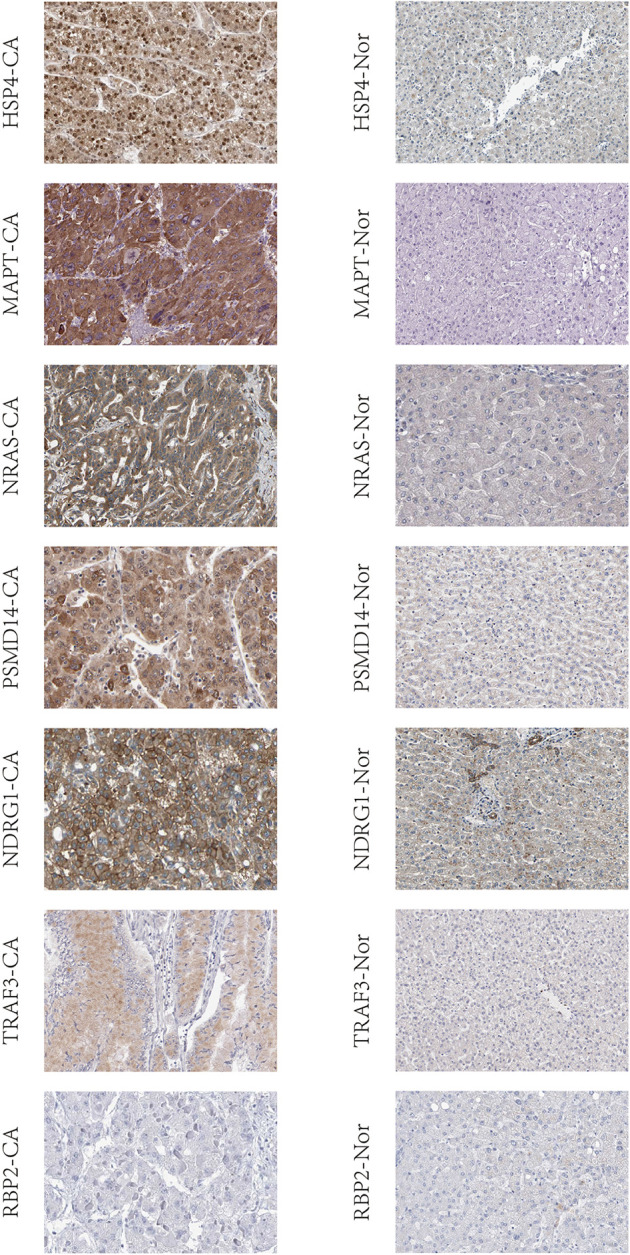
IRGs based on risk score were validated in liver tissue using human protein atlas database. CA: the hepatic tissue of liver cancer patients, Nor: the hepatic tissue of non-liver cancer patients.

The expression of IRGs that make up the risk score showed a significant positive correlation with patient prognosis and related clinical information. We further evaluated whether the expression of these IRGs was different between normal individuals and liver cancer patients. The protein expression of these genes in HCC using human protein atlas database. However, expression of these immune genes was remarkably higher in the hepatic tissue of liver cancer patients compared to non-liver cancer individuals (Figure 9).

## Discussion

There is increasing evidence that expression of immune genes is closely associated with the occurrence and progression of tumors (11, 12). Consequently, immunotherapy is now commonly used against various cancer types (13, 14), including HCC (15). Providing patients with a suitable treatment plan has become the core element of treatment (16). Although immunotherapy has been used to treat HCC, nevertheless, not all patients benefit from it. Thus, it is essential to develop an IRG-based score that can accurately predict the response to cancer immunotherapy. This will allow the most appropriate patients to be selected for treatment. Currently, several IRGs have been used to assess whether immunotherapy may be suitable and to predict the outcome of patients (17).

In our study, eight IRGs (HSPA4, PSMD14, RBP2, MAPT, TRAF3, NDRG1, NRAS and IL17D) with prognostic value in HCC patients were identified by Cox regression analysis and LASSO regression modeling. These IRGs, which were used to construct a risk score to evaluate the prognosis of HCC patients, showed significant positive correlations with HCC patient grade, AJCC stage and TNM. In other words, a high risk score correlated with worse patient prognosis. PSMD14 expression was significantly up-regulated in HCC tissues and was significantly associated with vascular invasion, tumor number, tumor recurrence and poor overall survival in HCC patients (18). High levels of NDRG1 expression correlated with poor prognosis in HCC patients (19), while NRAS overexpression was also associated with poor survival in HCC patients and proliferation *in vivo* (20).

HSPA4 expression was positively correlated with the grade of liver cancer in our study. Previous studies have shown that HSPA4 was overexpressed in liver cancer and was involved in tumor migration, invasion, transformation and recurrence (21, 22). TRAF3 has been reported to promote the proliferation of lung adenocarcinoma (23), but has not been well studied in liver cancer. Recent work has shown significant improvements in obesity, insulin resistance, liver steatosis and inflammatory responses in liver-specific TRAF3 knockout mice (24). Conversely, transgenic mice that overexpress TRAF3 in liver cells showed increased obesity. We found here that TRAF3 expression correlated significantly with the stage and TNM of HCC patients, suggesting it may be an important factor in the development of HCC.

Inflammatory processes contribute to immune tolerance as well as to tumor progression and metastasis. The role of IL17 in carcinogenesis, tumor metastasis and resistance to chemotherapy in several solid cancer types (25), together with high expression of IL17 in colorectal cancers, indicates an association with poor prognosis (26). Our results showed that IL17 expression correlated with tumor grade in HCC patients and that its expression was also significantly higher in female patients compared to males. IL17-mediated inflammation shows gender differences in a variety of diseases, including allergic asthma and urinary tract infection (27); to our knowledge, however, this has not been reported in liver cancer patients. HSPA4 was positively associated with immune cell infiltration and immune checkpoints (PD-1 and CTLA-4) in HCC (28). We validated the value of HSPA4, TRAF3 and IL17 in the diagnosis and prediction of prognosis of HCC. These proteins may be involved not only in the occurrence and development of HCC, but in its immune regulation as well. Therefore, they can be a potential diagnostic and prognostic biomarker as well as a therapeutic target for HCC. Although direct associations between HCC and the RBP2 and MAPT genes have yet to be reported, we believe these potential associations warrant further experimental study.

Furthermore, the high-risk group had higher proportions of macrophages, regulatory T cells (Tregs) and Th2 cells, whereas the activity of type II IFN responses, Mast cells and NK cells were lower in the high-risk group, which was associated with impaired antitumor immunity. Tumor-infiltrating macrophages are one of the most abundant stromal cell types in the HCC tumor microenvironment, not only suppressing anti-tumor immunity by inducing extracellular matrix remodeling, angiogenesis, tumor metastasis, and therapy resistance, but also secreting various inflammatory mediators that promote tumor cell progression (29). Tregs impair cancer immune surveillance by creating an immunosuppressive environment that promotes tumor cell survival (30). Disturbed immune response caused by up-regulation of Th2 cytokines and down-regulation of Th1 cytokines is a key predictor of HCC metastasis (31). The activity of type II IFN response, together with reduction of Mast and NK cells, was significantly associated with tumor immune escape and poor immunotherapy efficacy (32, 33). Therefore, weakened antitumor immunity in high-risk patients may be the key factor for the poor prognosis.

The tumor microenvironment coexists and interacts with a variety of immune cells to maintain the growth of HCC (34). The IRGs screened in the present study play an important role in the immune response to HCC. We evaluated the relationship between risk score and different types of immune cells and found an association with macrophages and neutrophils, in particular. The risk score developed herein was found to be valuable for predicting the outcome of HCC patients. Eight clinically significant IRGs in HCC were screened using bioinformatics, helping to expand our knowledge of the molecular mechanisms relating to HCC immunology. One of the limitations of our study was the small number of patients included in the validation group. We suggest that more HCC patients should be investigated in prospective studies to further understand the underlying mechanisms. In conclusion, we have established a valuable prognostic risk score for HCC patients, which may serve as a more accurate predictor of survival than the present method. The risk score's improved predictions for immune cells and functions can provide clinical guidance for the diagnosis and prognosis of different immunophenotypes, and provide multiple therapeutic targets for the treatment of HCC patients based on subtype-specific immune molecules.

## Data Availability

The original contributions presented in the study are included in the article/Supplementary Materials, further inquiries can be directed to the corresponding author/s.
